# Can a workplace dialogue impact the perceived influence of neck and/or backpain on everyday activities and performance at work? A secondary analysis from the randomized controlled trial WorkUp

**DOI:** 10.1186/s12891-022-05812-w

**Published:** 2022-09-15

**Authors:** Iben Axén, Charlotte Post Sennehed, Frida Eek, Kjerstin Stigmar

**Affiliations:** 1 Unit of Intervention and Implementation Research for Worker Health, Institute of Environmental Medicine, Karolinska Institutet, Nobels v. 13, S- 171 77 Stockholm, Sweden; 2 Department of Clinical Sciences Lund, Orthopedics, Lund University, Remissgatan 4, 22185 Lund, Sweden; 3 Department of Research and Development, Region Kronoberg, Sigfridsvägen 5, 35257 Växjö, Sweden; 4 Department of Health Sciences, Lund University, Margaretavägen 1b, 222 40 Lund, Sweden

**Keywords:** Work ability, Workplace dialogue, Neck and back pain, Sick leave, Primary care

## Abstract

**Background:**

Neck- and back- pain are highly prevalent conditions in Sweden and world-wide. Such pain often has consequences on everyday activities, work- and personal life. One consequence is work absence and decreased productivity at work. Adding a workplace dialogue to structured physiotherapy was recently found to lead to increased workability, i.e., not being on sick leave during the 12^th^ month of follow up.

**Aim:**

The aim of the study was to explore the effect of a workplace dialogue intervention on secondary outcomes: perceived impact of neck and/or back pain on everyday activities and on performance at work, and total days of sick leave during 12 month follow up. A further aim was to examine associations between perceived influence of pain, and sick leave.

**Method:**

Patients with neck and/or back pain in primary care in the south of Sweden were randomized into structured physiotherapy alone (*n* = 206) or with the addition of a workplace dialogue (*n *= 146). Data regarding the pain’s influence on everyday activities and on performance at work were collected using weekly text messages for 52 weeks. The pattern of change in perceived influence of neck and/or back pain on everyday activities and performance at work was compared between the groups with linear mixed models. Cross sectional correlations between perceived influence of neck and/or back pain on everyday activities and performance at work, and days of sick leave, during the preceding four weeks at months 3, 6, 9 and 12 were examined.

**Result:**

We found no differences in change of perceived influence of neck and/or back pain on daily activities or perceived performance at work, or total days of sick leave during the 12 months of follow up between the groups with structured physiotherapy with or without a workplace dialogue. There was a weak to moderate positive correlation between days of sick leave and perceived influence of neck and/or back pain on everyday activities and performance at work (rho 0.28–0.47).

**Conclusion:**

A workplace dialogue was not found to affect the perceived impact of neck and/or back pain on everyday activities and performance at work.

**Trial registration:**

ClinicalTrials.gov ID: NCT02609750.

## Introduction

Neck and back pain are conditions that are considered global burdens [1], as these conditions are highly prevalent, and often run an episodic course with remissions and exacerbations [2]. Whether in an acute episode or in a persistent course, the consequences of these conditions may affect the individual, their family, friends and colleagues, employers, and society. Neck and back pain lead to activity limitations and restrictions both at work and also in private life, such as caring for children and household chores such as sports and participation in social life [3, 4]. The interaction of individual factors and contextual factors determine these effects in each individual [5].

An important contextual factor is work. Most will prioritize their work despite not being well [6], so-called presenteeism, which often results in reduced performance at work compared to working when healthy [7]. A recent study investigated productivity among academics and found that the productivity loss for persons with health related problems was lower for/among persons with high job motivation and commitment [8]. Thus, performance at work may be affected negatively before a person takes sick leave [9]. Clinically, we see a succession of events: the first thing to be restricted by pain, is everyday activities, the second is work performance. Then, if pain persists, there are no other options than to go on sick leave.

The costs associated with neck and back pain are related to the individual, their employers and society [10]. For the individual, loss of income relating to sick leave may be a major burden. For the employer, reduced performance at work and staff replacements may result in increased costs when an employee is affected by neck and back pain.

For society, cost for interventions (investigations and treatment) may ensue, but productivity loss, absenteeism and presenteeism is by far the highest cost consequence of pain [11]. In Sweden alone, low back pain was estimated to be 740 million euro in 2011 [12].

Work is considered to be an important “wellness-factor” for the individual in terms of economic independence, psychosocial needs and individual identity [13], and keeping individuals with musculoskeletal pain at work is a priority in order to minimize health loss [14]. Work tasks may have to be modified and adjusted, which, in addition to decreased performance at work, may be costly for the employer. However, sick leave is still the costliest option for both employee and employer [15], and it seems the best solution to keep employees at work.

Some interventions have been explored directed towards returning to work for individuals with neck and back pain after being on sick leave [16]. Normally, these conditions are managed in primary care, but the workplace itself has been found to be important in this process [17].

In the WorkUp trial we investigated the effect of a workplace dialogue called Convergence Dialogue meeting (CDM) as an add on to structured physiotherapy in primary health care, for patients with sub-acute/acute neck and/or back pain [18]. The CDM encompasses three dialogues: one with the employee/patient, one with the employer and finally, one dialogue with the employee/patient and employer together. In this trial the PT took initiative to the CDM and was leading the discussion. The aim of the CDM was to find out about possible adjustments that could contribute to stay at work or return to work. The intervention was found to improve work ability, defined as no sick leave days during the last consecutive 4weeks of the 12-months follow up [18], compared to standard care and was also found cost-effective [19]. Self-reported outcomes (work ability, function and health related quality of life) were studied in a secondary analysis [20] but no differences were found between the intervention and reference groups.

The aim of this secondary analysis was to examine the effect of CDM on the perceived impact of neck and/or back pain on everyday activities and on performance at work. As we suspect there is a succession order (pain affecting everyday activities first, performance at work second and sick leave last), we expected that the intervention had a positive effect on the perceived impact of neck and/or back pain on everyday activities and on performance at work compared to structured physiotherapy only (the reference group). We also aimed to investigate the association between the perceived impact of neck and/or back pain on everyday activities and performance at work, and sick leave.

## Method

The aim of the study was to evaluate the effect of CDM added to physiotherapy care on the perceived influence of neck and/or back pain on [1] daily activities, and [2] performance at work, for individuals with neck and/or back pain. Further aims were to compare total number of sick leave days during the year following the intervention between the groups, as well as to examine cross sectional associations between sick leave and perceived influence of pain on daily activities and performance at work.

### Design

Data from this study stem from the WorkUp trial [18], which was a pair-wise cluster-randomized controlled trial in primary care (ClinicalTrials.gov ID: NCT02609750). The trial was approved by the regional ethics committee in Lund: Dnr 2012/497 (September 28, 2012), Dnr 2012/648, (October 30, 2012), and Dnr 2012/833 (January 9, 2013). The trial is described in detail in previous publication [18].

### Participants

Patients, 18–67 years of age with acute or subacute (< 12 weeks) neck and/or back pain were eligible for inclusion. Further inclusion criteria were no current sick leave, or no more than 60 days of sick leave the previous year had been working at least four consecutive weeks the last year and (were considered at risk for sick leave by)scoring 40 points or more at “Örebro Musculoskeletal Screening Questionnaire (short version) [21]. In total, 352 patients were included from January 2013 through December 2014.

### Setting

Primary care centers in Region Skåne, Region Kronoberg and Blekinge county council in the south of Sweden.

### Interventions

Twenty health care units were pair-wised cluster-randomized to reference or intervention groups. Patients received structured physiotherapy (reference) or structure physiotherapy plus the CDM-intervention (intervention). The CDM consisted of three meetings; one with the patient, one the employer and finally all together [18], and centered around a discussion about workplace adjustments to facilitate work despite the neck and/or back pain.

### Data collection

Participants in the WorkUp-study answered a number of questionnaires at baseline regarding gender, age, marital status, education, employment, sick leave and health related quality of life. All participants were offered visits to the physiotherapist for follow-up examinations and answered questionnaires at 3, 6 and 12 months after baseline.

In the trial, patients also answered weekly short text messages (SMS) for one year. They were asked about number of days on sick leave, and to rate to what extent they perceived their pain to influence their everyday activities and their performance at work.

The SMS questions were:

- 1: How many days were you on sick leave the past week? Reply with a number between 0 and 7.
- 2: During the past week, to what extent did your neck and/or back problems affect your performance at work? Reply with a number between 0 and 10 (0 = no effect, 10 = stopped me completely).
- 3: During the past week, to what extent did your neck and/or back problems influence your ability to perform everyday activities (home, leisure)? Reply with a number between 0 and 10 (0 = no effect, 10 = stopped me completely).

As previously reported [18] the response rate of the SMS never dropped below 84% during the 12 months of the trial and was similar in the intervention and the reference groups. Answers to the SMS-questions were automatically entered into a spreadsheet, available for analysis.

### Outcomes

In this study, secondary outcomes from the trial were analyzed; perceived influence of neck and/or back pain on everyday activities and on performance at work. Furthermore, we compared the total number of self-reported sick leave days during the 12 months follow up.

### Sample size

Power was calculated based on the primary outcome: the number of sick leave days during the last 4 weeks of the 12 month-trial. A minimum of 20 clusters (primary care units) and 259 patients in each arm (518 in total) were required.

### Randomization

The 20 primary care units that expressed an interest in participating in the study were matched according to size (registered population), community size of the units’ location, patients’ morbidity; ACG- Adjusted Clinical Groups [22, 23] and socioeconomic status; CNI- Care Need Index [24]. An independent statistician used a computer-generated program to allocate the pairs (resulting in 10 intervention and 10 reference primary care units).

### Procedure

Patients meeting inclusion criteria were included consecutively after being informed about the study. They all signed informed consent forms.

Based on participants’ individual needs, contacts with medical doctor, psychologist, or occupational therapist were initiated. Care was individualized regarding content and duration and included examination, assessment, diagnosis, evidence-based treatment and follow-up in both groups according to each patients’ needs. The patients could discuss issues relating to their pain and get advice if needed.

### Statistical analysis

The pattern of change of perceived influence of neck and/or back pain on daily activities and performance at work was analyzed with two separate general linear mixed models. The models were solved using the restricted maximum likelihood (REML) method. Independent variables were *group* (intervention or control), and *time* (five time-points: baseline, 3, 6, 9 and 12 months). Dependent variables were the average perceived influence of neck and/or back pain on 1) daily activities and 2) performance at work, over the recent four weeks preceding each follow up point (3, 6, 9 and 12 months). Main effects of group and time, as well as the two-way interaction *group* by *time*, were included in the models. The Schwarz Bayesian Information Criterion was used to guide the final selection of covariance structure. For both analyses, first order autoregressive (AR1) covariance structure was applied. In case of significant interaction effects, changes from baseline to each follow up time point were calculated and compared between groups through analysis of variance (ANOVA), adjusted for baseline values. The total number of sick leave days during the year of follow up was summarized for each individual. Due to skewed distribution, the comparison of number of sick leave days between groups were performed with Mann–Whitney U test and presented as median (md) and inter quartile range (IQR); Q1-Q3. The association between number of self-reported days of sick leave and average perceived influence on 1) daily activities and 2) performance at work, during the preceding four weeks, was examined at each follow up point (3, 6, 9 and 12 months) with Spearman correlation.

All analyses were performed in SPSS (version 27). Alpha level was set to 0.05.

## Results

The inclusion period was planned for 12 months. Even though we had 20 participating units including patients, we needed to extend the inclusion period to 24 months due to slow enrolment. The trial ended up in 352 included patients and was underpowered [18].

Descriptive information of the 352 participants in this trial is summarized In Table 1. In short, the sample consisted of 2/3 women, largely below the age of 50. The primary reason for consulting was low back pain (nearly 70%) followed by neck pain (around 20%).

**Table 1:** Baseline data for the intervention and reference groups in the WorkUp-trial

Variable	Intervention *n* = 146	Reference *n* = 206
Age in years, mean (SD)	43.8 (± 11.6)	43.7 (± 12.6)
Sex, female, % (n)	63 (92)	67 (138)
Education, % (n)		
Primary School	11.0 (16)	6.8 (14)
Upper Secondary School	47.3 (69)	51.9 (107)
University > 3yrs	19.2 (28)	23.8 (49)
Other	22.6 (33)	16.9 (35)
Diagnosis, % (n)		
Cervicobrachial	18.5 (27)	23.8 (49)
Lumbago-sciatica	69.9 (102)	68.0 (140)
Cervical + lumbar	6.2 (9)	5.3 (12)
Myalgia	5.5 (8)	2.4 (5)
Sick leave, % (n)	34.9 (51)	35.9 (74)
If yes, 100%, % (n)	78.4 (40)	83.8 (62)

### Compliance with the CDM intervention

The CDM intervention was intended to consist of three meetings between the physiotherapist, the employee (the patient) and the employer. The extent to which the intervention was delivered as intended, is shown in Table 2 below.

**Table 2:** Frequency of delivered components in the CMD intervention for the intervention group (*n *= 146)

Component	n	%
No first interview	7*	4.8
First patient interview	48	32.9
Employer interview, 1 + 2	31	21.3
Three-party talk, 1 + 2 + 3	60	41.1

The full intervention is 1 + 2 + 3, all three steps

^*^7 participants were unable to receive any of the intervention steps, due to their studies/jobs

### Perceived influence of neck and/or back pain on daily activities

**Fig. 1:**
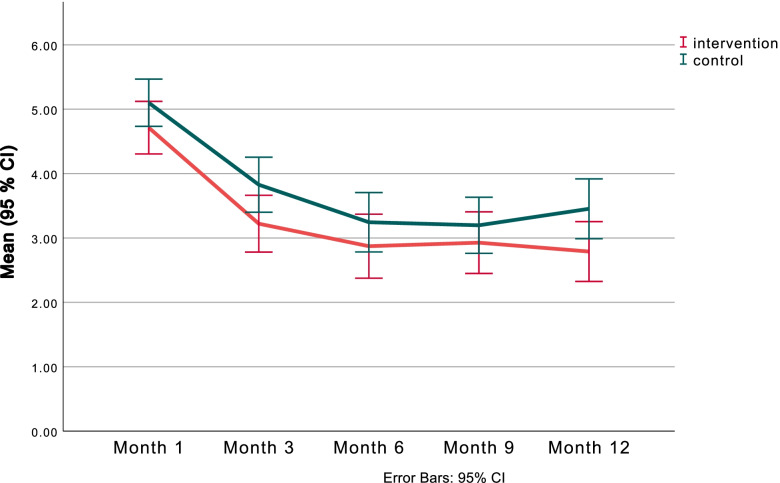
Change over time in self-reported perception on how neck and/or back pain affected daily activities, in the intervention- (red) and control (green) groups. (Scale 0–10 where 0 = no effect and 10 = stopped me completely)

The perceived influence of neck and/or back pain on daily activities (Fig. 1) decreased from baseline (main effect of time *p *< 0.001). There was a significant decrease at all follow up time points compared with baseline, as well as at 6, 9 and 12 months compared with 3 month follow up (*p* < 0.003)). The change over time followed a parallel pattern for the two groups (Fig. 1), with no significant main group differences (*p* = 0.128) or interaction effect between group and time (*p* = 0.203).

### Perceived influence of neck and/or back pain on performance at work

**Fig. 2:**
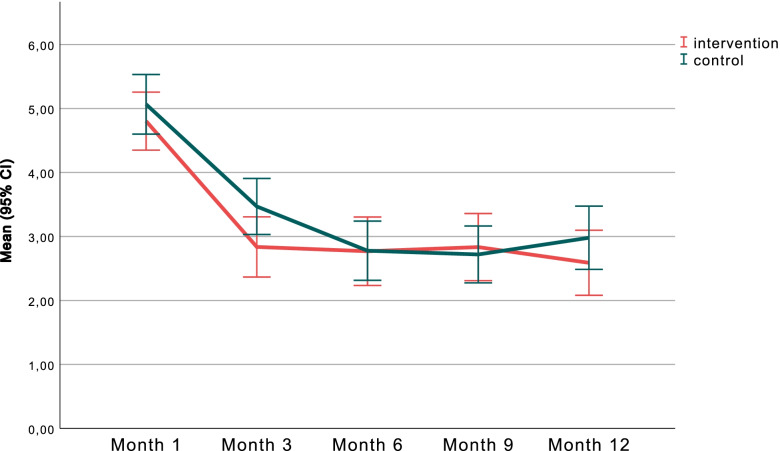
Change over time in self-reported perception on how neck and/or back pain affected performance at work, in the intervention- (red) and control (green) groups. (Scale 0–10, where 0 = no effect and 10 = stopped me completely)

The perceived influence of neck and/or back pain on performance at work (Fig. 2) decreased from baseline (main effect of time *p* < 0.001); all follow-up time points differed significantly from baseline, and there was also a significant decrease from 3 month to 6, 9 and 12 months (*p* = 0.033), while there were no significant main group differences (*p* = 0.33). A significant interaction effect between group and time (*p* = 0.029) indicated a different pattern of change between the groups. There were however no statistically significant group differences regarding change from baseline to each follow up point (*p* = 0.19).

### Days of sick leave

There was no significant difference regarding total number of days of sick leave during the year of follow up between the intervention group (md 22 (IQR 6–52)) and the reference group (md 18.5 (IQR 3–53.25) (*p* = 0.264).

### Cross sectional associations between sick leave and perceived impact of neck and/or back pain on daily activities and performance at work

The correlations between days of sick leave during a month and perceived influence of neck and/or back pain on everyday activities and performance at work were weak to moderate (Daily activities: Rho 0.283–0.395, *p* < 0.001, performance at work: Rho 0.326–0.465; *p* < 0.001;) (Table 3).

**Table 3:** Correlation (Spearman’s rho) between days of sick leave during a month and perceived influence of neck and/or back pain on everyday activities and performance at work during the preceding four weeks

Days of sick leave during the	Average perceived influence on everyday activities	Average perceived influence on performance at work
3rd month	0.395**	0.465**
6th month	0.283**	0.334**
9th month	0.349**	0.414**
12th month	0.290**	0.326**

^**^ *p* < 0.001

## Discussion

### Summary

No statistically significant differences in perceived influence of neck and/or back pain on daily activities or perceived performance at work were found between the groups with structured physiotherapy with or without a workplace dialogue. Thus, our expectation that such pain’s influence on leisure time activities and performance at work were affected by CDM, was refuted. Further, no difference in the total days of sick leave during the following year was found between the groups. There was a weak to moderate correlation between perceived influence of neck and/or back pain on everyday activities and performance at work, and days of sick leave.

### Discussion of results

We expected a “succession order” due to clinical observation, that the CMD-intervention would positively affect the perceived impact of neck and/or back pain on everyday activities and performance at work, as it was previously shown to affect work ability at 12 months follow-up [18]. The results did however not show any significant differences in outcome between the intervention and control groups. The intention of the WorkUp trial was to find employees at risk of sick leave and to use the CMD intervention to keep them at work despite their neck and/or back pain, or if on short-term sick leave, support a fast return to work. In hindsight we may conclude that, as CMD is designed to target the workplace, the potential for affecting performance at work as well as on other dimensions of the pain experience may be limited. In the intervention, both the patient and the employer were asked if they considered that back and/or neck pain was due to work conditions or conditions outside work. This opened up the possibility to address factors outside work. Unfortunately, we do not have any information regarding the answers to these questions or the agreements that followed. A previous secondary analysis from the WorkUp trial failed to show long-term effects of the intervention on self-reported outcomes workability (based on the Work Ability Score), function (based on Functional Rating Index) and health (based on the EuroQol 5 dimensions) [20]. The total number of days on sick leave during the following year was also similar between the groups.

Everyday activities include all the aspects that are necessary for a functioning life and has been shown to be affected by pain as well as to be associated with work limitations [25]. In a recent narrative review, workplace modifications were one of the identified factors with a positive influence on every-day activities [26]. In this study, we could not confirm that CDM changed the perceived influence of neck and/or back pain on everyday activities.

Performance at work was, in this study, experienced by the employee. It may contain a quantitative component: serving a certain number of clients, teaching a certain number of classes etc., but there is also a qualitative component concerning *how* the work was performed, i.e., the quality of the work. An individual may have been able to deliver the same amount as usual, but not with the same effort or precision. Musculoskeletal pain has previously been found to affect performance at work and has been suggested as an appropriate avenue for sustaining good work performance [27].

Physiotherapists in Sweden often address work-related aspects of their patients’ pain, but structured approaches regarding performance are, to our knowledge, seldom used. Contacts with the employer are often discussed when a patient is on sick leave, but traditionally the medical doctors or the occupational health services have these dialogues.

The physiotherapists involved in this study received training to conduct the CMD intervention, and they needed to build up good communication skills and authority over time, with practice. They were instructed to talk about work as part of the CDM intervention but did not discuss performance specifically and were not expected to present solutions like workplace adjustments, as physiotherapists in primary health care are not generally experienced in this area. Patients participating in the intervention experienced few concrete suggestions regarding workplace adaptations, but reported good support from their physiotherapist [28].

The intervention may also have been challenging for the employers. One assumes that there is a motivation to keep employees at work and thus a willingness to help this process. However, there may have been practical or economical constraints. Obviously, if few adjustments were made, the effect may be underestimated. The agreed workplace adjustments and modifications were recorded, but not followed up. Furthermore, the reference group may have had similar adjustments as part of their care, even without a workplace dialogue, which would also minimize the difference between groups [29]. Forsbrand et al. also reported that 54% of the reference group (compared to 82% of the intervention group) received similar adjustments as part of their care, even without CDM [29]. Structured physiotherapy could also include ergonomic advice, but patients in the intervention group received more such advice (81.7% compared to 54.2% in the reference group, *p* = 0.001) (not published results). This could be an explanation for the small differences between the groups.

The total number of days of days of sick leave was not found to be different between the reference and intervention groups during the 12 month follow up. This is contrast to the results in the main study WorkUp [18] where the result showed improved work ability defined as working at least 4 consecutive weeks at 1-year follow-up. This late effect may be because workplace adjustments is a process that takes time to implement. As discussed herein, the CDM method requires more education, time and support to the involved parties, including a focus on the ability to perform well at work and in everyday activities.

Weak to moderate correlations between the number of days on sick leave and the perceived impact of neck and/or back pain on everyday activities and performance at work were found, which indicates that a potential reduction in the perceived influence of neck/back pain on everyday activities and performance at work also could have reduced the number of sick leave days. However, in the current study we could not find any effect on either the perceived influence of pain on these parameters, or on days of sick leave.

### Strengths and weaknesses

This study was based on data from a randomized controlled trial, with a successful randomization. The outcome data were collected using simple questions in frequent text messages, which rendered a high response rate. Self-reported sick leave data has the advantage of covering all days of absence, while Swedish registry data of sickness absence does not include periods of leave of less than 14 days.

In this study, more than 62% of the patients in the intervention group received at least two steps of the CDM, but what was discussed in the meetings or if the dialogue resulted in any workplace adjustments is unknown. At the time of the trial, involving employers in discussions about employees in need of such adjustments was not standard practice, and initiating and leading a workplace dialogue was not something most physiotherapists normally did. Thus, the CMD-intervention may have been experienced as challenging for many of the participating physiotherapists.

The sample size calculation of the trial was based on the primary outcome; work ability, defined as not being on sick leave in the last 4 weeks of the 12 months intervention. Despite prolonging the inclusion period, the trial was underpowered. However, the primary results still found a significant difference between groups in the primary outcome. In our study though, also the observed pattern was quite similar between the groups.

Another weakness of the study is the unknown non-inclusion of participants. Even though inclusion was intended to be consecutive, clinical routines sometimes made this difficult, and some possibly eligible patients were never asked to participate. No records were kept of these individuals, so a comparison with the included subjects is not possible to make.

The short text messages questions used to gather data in the trial were carefully constructed, to make them short and easy to understand. They have not been validated against other instruments, so we do not know how the participants perceived them. Possibly, the responses are not answering the intended measures. For instance, the question about work performance may not have been clear to the participants, i.e., the concept of work quality may not be something people generally consider. This concept requires a certain ability to abstract, in contrast to counting the number of days on sick leave. Thus, we cannot be sure that the answers were pertaining to the intended measure. However, the SMS-system allows for a continuous communication between the researcher and the participant, and very few questions were asked about the interpretation of the SMS questions.

### Generalizability

The population in the southern part of Sweden are very similar to the rest of the country, and care was taken to include diverse primary care units with geographical spread. The included primary health care units were matched in pairs and represented a diversity regarding CNI [24] and ACG [22, 23]. The cluster-randomization minimized contamination between interventions. However, it was difficult to reach power, and along with not recording non-invited subjects, no recording of subjects that declined participation was done, making an assessment of generalizability difficult.

## Conclusion

In this secondary analysis of a cluster-randomized controlled trial with 12-months follow-up, we found no evidence that the CDM intervention affected the perceived influence of neck and/or back pain on everyday activities or performance at work, or total number of sick leave days during the follow up. This contrasts with the results of primary outcome, where more patients in the intervention group were found to have work ability, defined as no days of sick leave during the 12^th^ month of follow up. There was however a weak to moderate association between perceived impact of neck and/or back pain on everyday activities and performance at work, and days of sick leave. The relationship between functional limitations such as pain, performance at work and being sickness absent or not needs to be further studied.

## Data Availability

The ethical permission does not allow sharing of data. The principal of the study is Region Skåne, together with Region Kronoberg and Region Blekinge. The outputs of the analysis for the current study are available from the corresponding author on reasonable request.

## Abbreviations

CDM: Convergence dialogue meeting
SMS: Short message service (text message)
ACG: Adjusted clinical groups
CNI: Care need index
REML: Restricted maximum likelihood
ARI: First order autoregressive covariance structure
ANOVA: Analysis of variance
IQR: Interquartile range
SPSS: Statistical package for the social science
SD: Standard deviation
